# Rationale for constant flow to optimize wastewater treatment and advanced water treatment performance for potable reuse applications

**DOI:** 10.1002/wer.1531

**Published:** 2021-02-15

**Authors:** George Tchobanoglous, John Kenny, Harold Leverenz

**Affiliations:** ^1^ Department of Civil and Environmental Engineering University of California Davis CA USA; ^2^ Trussell Technologies, Inc. Oakland CA USA

**Keywords:** advanced water treatment, constant flow wastewater treatment, divided and satellite treatment, flow equalization, pathogen log removal credits, potable reuse, process optimization

## Abstract

Population growth, the impacts of climate change, and the need for greater water security have made the reuse of wastewater, including potable use, increasingly desirable. As interest in potable reuse of wastewater increases, a variety of processes have been proposed for advanced water treatment following conventional wastewater treatment. In all cases, the operation and performance of advanced water treatment facilities (AWTFs) is improved when the treated wastewater feed is of the highest quality that can be achieved and the advanced water treatment (AWT) processes are operated at a constant flow. One proven method of optimizing the performance of wastewater treatment facilities (WWTFs) is constant flow operation with no extraneous return flows other than internal process recycle flows, such as return settled solids. A number of approaches can be used to achieve constant flow including flow equalization, divided treatment trains, and satellite treatment. The ways in which constant flow wastewater treatment benefits both WWTFs as well as the AWTFs are considered with special emphasis on the ability to achieve predictable log removal credits (LRCs) for specific microorganisms. Actual performance data from constant flow WWTFs are used to illustrate how LRCs are determined.

**Practitioner points:**

- Constant flow WWTFs should be considered to produce the highest quality secondary effluent for AWT.
- Flow equalization, divided treatment trains, and satellite treatment can be used to achieve constant flow to optimize wastewater treatment in small and medium size WWTFs.
- Flow equalization can be used to maximize the amount of wastewater that can be recovered for potable reuse.
- Important benefits of constant flow for wastewater treatment facilities include economic and operational savings, stable and predictable treatment performance, energy savings, ability to optimize performance for the removal of specific constituents, and the ability to assign pathogen log removal credits (LRCs).
- Important benefits of constant flow and optimized WWT for AWTFs include economic and operational savings; less pretreatment needed, including energy and chemical usage; elimination of the need to cycle treatment processes; and added factor of safety with respect to the required pathogen LRCs.
- In large WWTFs, constant flow for AWTFs will typically be achieved by effluent diversion; depending on the effluent quality additional pretreatment may be needed.
- The design and implementation of WWTFs and AWTFs for potable reuse should be integrated for optimal performance and protection of public health.

## Graphical abstract

Methods of achieving constant flow in WWTFs for potable reuse: (a) use of satellite WWTFs, (b) use of flow equalization facilities, and (c) use of divided WWTFs. In very large treatment facilities constant flow can be achieved by diverting a fixed portion of the treated effluent.

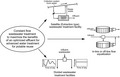

## Introduction

interest in potable reuse is increasing throughout the world, as the amount of available fresh water has remained the same or has, in some cases, decreased, while the population dependent on the available water has grown. The situation is especially serious for coastal communities where seawater intrusion impacts groundwater supplies and treated wastewater, which could be reused, is instead discharged, e.g., to estuaries and the ocean. Using water only once before discharging it as waste will not be sustainable for most large cities. Potable reuse represents an opportunity to reduce demand on available fresh water and to manage water resources more effectively. There are two types of potable reuse: indirect and direct, with the principal difference being that an environmental buffer is employed in indirect potable reuse (Tchobanoglous et al., 2015). In both cases, secondary effluent from a wastewater treatment facility (WWTF) is processed further in an advanced water treatment facility (AWTF). To optimize the performance of the advanced treatment processes employed at an AWTF, the characteristics of the treated effluent from the WWTF should be predictable and of the highest quality that can be achieved.

A proven method of performance optimization for WWTFs is constant flow operation, with no extraneous return flows other than internal process recycle flows, when combined with a comprehensive source control program. The need for and benefits of comprehensive source control were discussed in a recent paper (Tchobanoglous & Leverenz, 2019). The focus here is on benefits of constant flow wastewater treatment as a precursor to advanced water treatment (AWT) for potable reuse. Discussion topics include flow and loading patterns at existing WWTFs; how constant flow can be achieved in existing and new WWTFs; the benefits that can be derived from constant flow for wastewater treatment; the benefits of constant flow for AWT; and illustration of the microorganism LRCs that can be achieved at two constant flow WWTFs.

## Flow and loading patterns at existing WWTFs

Flow and loading patterns observed at existing WWTFs can be characterized as (1) variable flow with variable constituent concentrations and mass loadings and (2) constant flow with reduced variability in the constituent concentrations and mass loadings. The benefits of constant flow operation are discussed in detail in the subsequent section.

### Variable flow and variable constituent loading

**Figure 1:**
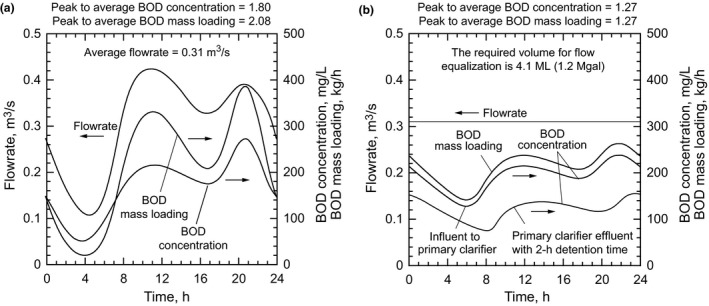
Typical hourly variation observed in wastewater flow, BOD concentration, and BOD mass loading: (a) un‐equalized values after screening and grit removal and (b) equalized values after in‐line flow equalization and BOD concentration following primary sedimentation. (Adapted, in part, from Tchobanoglous et al., 2014).

When considering the flow regime at most WWTFs, two different operating periods are important: dry weather and wet weather. During the dry weather periods, most WWTFs operate with an influent flowrate that varies as shown on Figure 1a. With water conservation, the variation in peak to average and peak to low flow observed in small [0.0440–0.44 m^3^/s (1–10 Mgal/d)] WWTFs has become even more pronounced. As a result of the COVID pandemic stay at home restrictions, a decrease has been observed in the peak to average flow ratio during the daytime hours, while the average to low flow ratio has remained about the same, especially at small and medium sized facilities (Enfinger & Stevens, 2021). The biochemical oxygen demand (BOD) concentration also varies throughout the day, resulting in a variable mass loading as illustrated in Figure 1a. A complicating factor, not reflected in the influent BOD concentration and mass loading values, is the impact of return flows from solids processing and dewatering operations. Typically, these flows are returned during daytime hours, when treatment capacity is limited. Thus, because of the variability of the input parameters and the presence of return flows, the reported effluent constituent concentrations are not uniform, but follow some statistical distribution.

**Figure 2:**
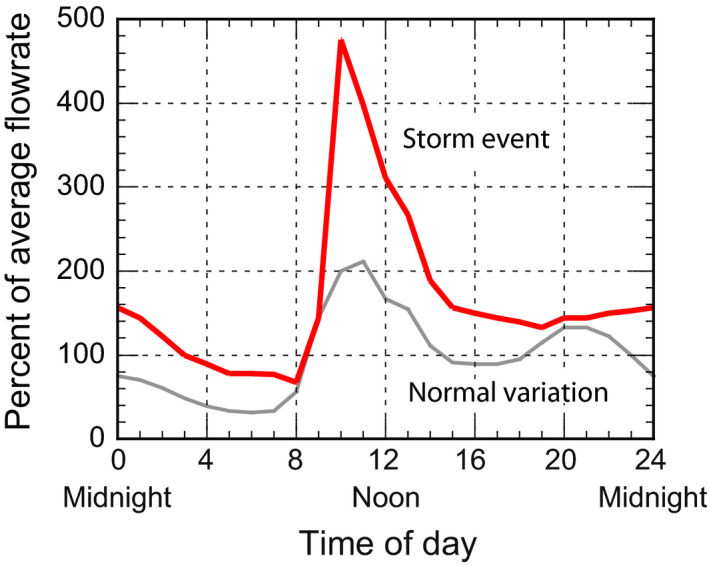
Observed flow rate variation over a 24‐h period under normal dry conditions and during a rainfall event.

During wet weather periods, the flowrate variation during storm events often increases significantly as shown in Figure 2. Historically, peak wet weather flowrates have often exceeded the average flowrate by a factor of five or more. With climate change, the intensity of rainfall events, during storm periods, has increased significantly with resulting peak to average flowrate ratios as high as 10 now being observed, depending on the condition of the collection system. With continuing water conservation, constituent loading variations similar to those for flowrates have also been observed. Such flowrate and loading fluctuations are not addressed adequately in conventional WWTF design. As a result, conventional WWTFs struggle to provide effective stable treatment of wastewater that is to serve as an influent to an AWTF (Plósz et al., 2009).

### Constant flow with reduced variability in constituent loading

**Figure 3:**
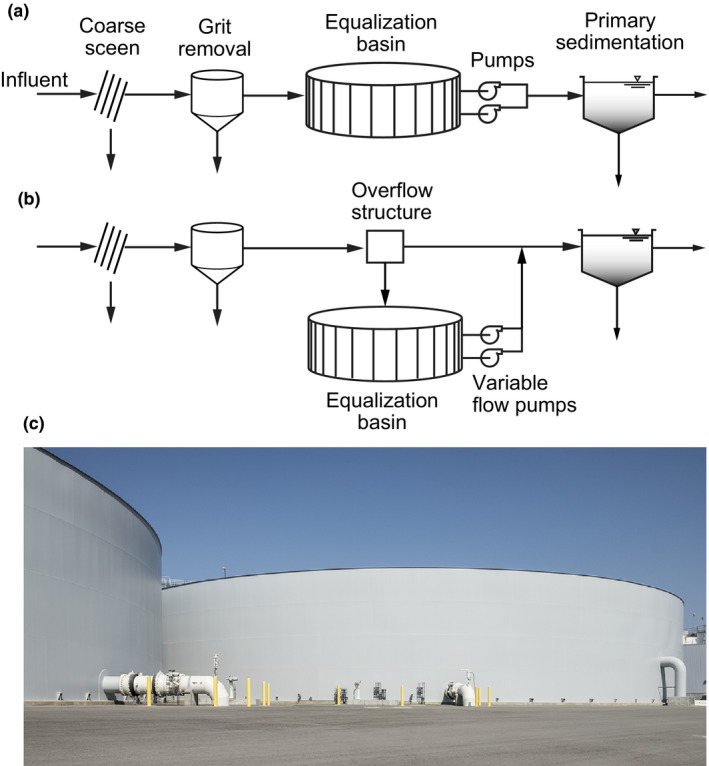
Definition sketch for (a) in‐line and (b) off‐line flow equalization and (c) view of two new in‐line 8.4 ML (7.5 Mgal) flow equalization storage tanks at Orange County Water District's Groundwater Replenishment System. When used in conjunction with available storage capacity available at the Orange County Sanitation District, 76 ML (20 Mgal) of flow equalization is available.

Constant flow operation can be realized by a variety of means including flow equalization. The various modes of achieving flow equalization are discussed in the following section. Where a flow equalization tank is inserted after coarse screening and grit removal [see Figure 3a] and before primary sedimentation, variations in BOD concentrations and mass loadings are reduced as shown in Figure 1b. If primary sedimentation is employed following flow equalization, the BOD concentration will be reduced further [see Figure 1b]. Even further BOD concentration reductions can be achieved if primary effluent filtration (PEF) is employed (Caliskaner et al., 2020). The benefits of flow equalization for WWT and AWT are considered following the discussion of means used to achieve constant flow.

## Achieving constant flow at WWTFs and AWTFs

Three planned methods for achieving constant flow in small [0.0440–0.44 m^3^/s (1–10 Mgal/d)] and medium sized [0.44–4.4 m^3^/s (10–100 Mgal/d)] WWTFs are by means of flow equalization, divided treatment trains, and satellite treatment. Before considering how constant flow is achieved in very large [>4.4 m^3^/s (>100 Mgal/d)] WWTFs it is useful to consider a phenomenon that has resulted from water conservation, namely *de‐facto* flow equalization.

While the focus of the following discussion is on achieving constant flow for more effective WWT and AWT, another important benefit of flow equalization at small and medium sized WWTFs is the potential to maximize the recovery of wastewater for potable reuse, especially where large diurnal flowrate variations occur. The economic benefits of flow equalization for the recovery of wastewater are considered in the section dealing with the benefits of constant flow for AWT.

### Constant flow through use of flow equalization

Two types of planned flow equalization facilities are used to achieve constant flow: in‐line or off‐line [see Figure 3a,b]. The choice of in‐line or off‐line equalization will depend on local conditions, the amount of flow and/or load equalization required, where these facilities are to be applied in the treatment process flow diagram, and what type of structure is used (e.g., lined earthen basins or engineered tanks). Typically, flow equalization is employed following coarse screening and grit removal and before primary sedimentation (see Figure 3). Alternatively, flow equalization facilities can be employed following primary sedimentation (Tchobanoglous et al., 2014). The actual physical location of the flow equalization facilities can be on‐ or off‐site. Although the implementation of flow equalization facilities will produce constant flow, the management of return flows remains an issue.

Return flows from sludge thickening, digestion, dewatering, and storage processes can have negative impacts on the performance of biological treatment processes. Typically, these flows are returned as produced to the head end of the WWTFs or introduced directly to secondary process for treatment, increasing the organic, nutrient, and colloidal loading, and, in turn, deteriorating treatment performance. For example, anaerobic and aerobic digestion results in the release of humic and fulvic acids, soluble organic nitrogen‐containing compounds, ammonium, and ortho‐phosphate into the bulk liquid. The presence of Mannich polymers, found in recycle streams from sludge thickening and dewatering operations, has been implicated in the formation of N‐nitroso dimethylamine (NDMA). Flow equalization and treatment before reintroducing return flows into the treatment process are the principal methods used to mitigate the impact of these flows. Sidestream treatment of these flows has been conducted at the Washington D.C. Blue Plains Advanced Wastewater Treatment Plant since 2011 (Figdore et al., 2018). Additional details on sidestream constituent concentrations and treatment processes may be found in Tchobanoglous et al. (2014)

### Constant flow through use of divided treatment trains

**Figure 4:**
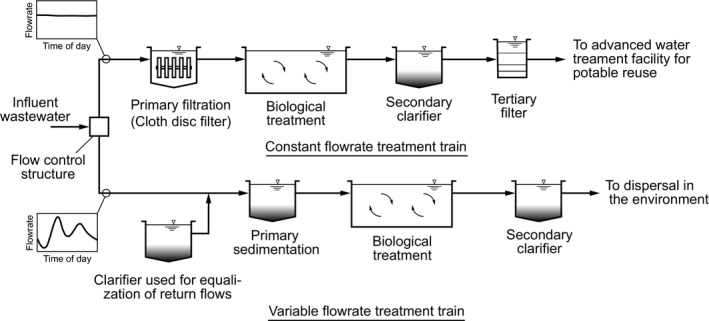
Definition sketch for the implementation of divided flow at an existing WWTF. Primary filtration is employed to reduce the influent total suspended solids (TSS) and organic loading to the constant flow WWTF (Adapted from Tchobanoglous & Leverenz, 2019; Caliskaner et al., 2020).

At existing WWTFs, where excess capacity is available as a result of water conservation and or over‐design, treatment facilities can be repurposed such that one part of the plant can be isolated for operation at constant flow with reduced constituent loadings to produce an effluent optimized for AWT (see Figure 4). Because primary facilities are often difficult to isolate, primary filtration (PF) with a cloth disk filter could be used for the isolated portion of the treatment plant (Caliskaner et al., 2020). Where large diurnal flows are experienced, excess primary sedimentation capacity can be used for flow equalization. Excess tankage can also be used for the treatment of return flows. Treated return flows can be discharged to either the constant or variable flow treatment trains, depending on the quality. Additional damping of constituent concentrations and mass loadings can be achieved if PF is used.

### Constant flow through use of satellite wastewater treatment facilities

**Figure 5:**
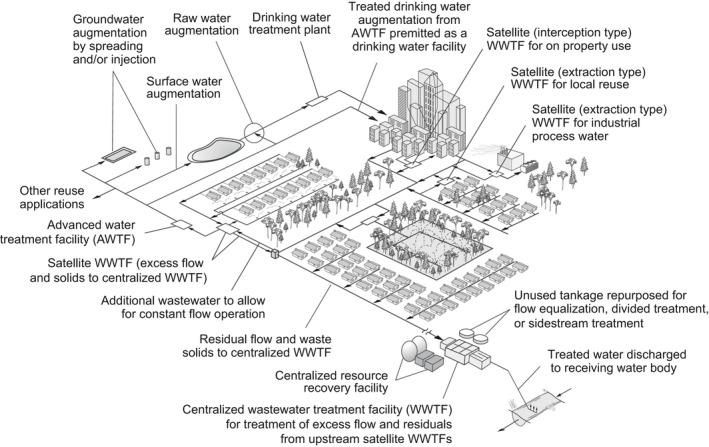
Integrated wastewater management system employing a satellite WWTF; an extraction type reuse systems where wastewater is extracted from a collection system, treated, and reused locally; and an interception system where the wastewater is intercepted before it reaches the sewer is treated and reused locally for toilet flushing and landscape irrigation (Adapted from Asano et al., 2007; Gikas & Tchobanoglous, 2009).

**Figure 6:**
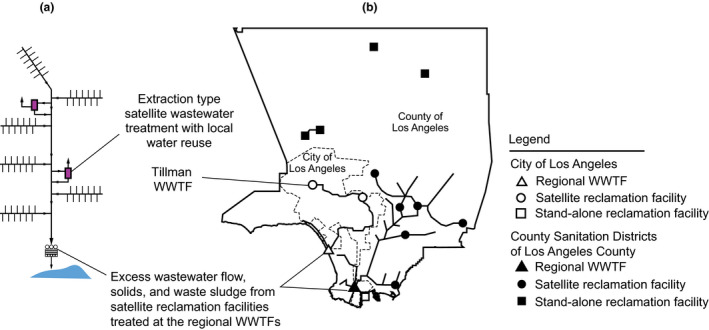
Integrated wastewater management system employing extraction type satellite constant flow WWTFs (a) definition sketch and (b) diagram of the satellite treatment systems employed by the City of Los Angeles and the Sanitation Districts of Los Angeles County (Adapted from Tchobanoglous et al., 2014).

Historically, centralized wastewater collection systems are arranged to route wastewater to remote locations for treatment near a dispersal location. Among the most feasible alternatives to achieve constant flow treatment is the use of satellite WWTFs, as part of an integrated water management system for both non‐potable and potable reuse applications. In an integrated water management system, satellite WWTFs, as illustrated on Figure 5, typically are located within the sewer service area at or near the point of waste generation and or potential reuse applications (Tchobanoglous, 2019). Satellite WWTFs and water reuse systems can take a number of forms, as illustrated on Figure 5, including (1) treatment facilities for subdivisions, portions of a community, or an entire community in a regional system; (2) extraction type treatment facilities, large and small, where wastewater is extracted from a wastewater collection system, treated, and used for specific local and regional reuse applications; and (3) interception type treatment facilities, used commonly for in‐building recycling, where the wastewater to be treated and reused is intercepted before reaching a collection system (Asano et al., 2007). In large collection systems, satellite WWTFs will, most commonly, be of the extraction type. Examples of extraction type satellite WWTFs are those operated by the City of Los Angels and the Sanitation Districts of Los Angeles County, as shown in Figure 6.

The most important feature of satellite WWTFs is that they can be operated with a constant flowrate. In some locations, because of the ongoing long‐term effects of water conservation, the amount of wastewater that can be extracted from the collection system is less than the design capacity of the satellite WWTF. Where the wastewater flow is inadequate, it may be necessary to supplement the available flow with wastewater from another sewershed or WWTF (the Tillman Water Reclamation Plant, operated by the City of Los Angeles is an example). In some locations, where a large residential area serves as the source of wastewater for a satellite WWTF and co‐located AWTF, incorporation of flow equalization in the design of the WWTF makes it possible to recover the maximum amount of wastewater despite the large diurnal variations (the North City Water Reclamation Plant in San Diego is an example). Further, because there are no solids processing facilities, there are no return flows. All of the solids including coarse screenings, solids removed by sedimentation or primary filtration, and excess solids from biological treatment, are returned to the collection system for treatment at a downstream centralized treatment facility. Another interesting aspect of satellite treatment is the potential for the control of corrosion in the collection system by discharging nitrified waste activated sludge (Beecher et al., 1959). The nitrate concentration could be supplemented with the addition of pure oxygen if required.

### Unplanned de‐facto flow equalization

In large collection systems, the diurnal flowrate variation is reduced because of the length and internal storage capacity of the extended collection system. With the reduction in wastewater flowrates brought about by water conservation, the excess collection system volume often serves as *de‐facto* flow equalization storage, damping flow variation and constituent concentrations and mass loadings. For an example of the effects of conservation on flowrates, the Hyperion Wastewater Treatment Plant serving the City of Los Angeles has a treatment capacity of 19.72 m^3^/s (450 Mgal/d) but currently treats about 12.71 m^3^/s (290 Mgal/d). Where holdup times are excessive, the organic constituents in the wastewater can undergo anaerobic decomposition, which alters the characteristics of the wastewater.

### Constant flow through effluent diversion

In large WWTFs where it may not be possible, needed, or economically feasible to achieve constant flow through the use of conventional flow equalization facilities, constant flow can be achieved by diverting a portion of the secondary effluent. While the benefits of flow equalized WWT may not be realized, the pretreatment facilities, if needed, and the AWTF will benefit from constant flow operation. As part of the Regional Recycled Water Program (RRWR) in Southern California, it is proposed to implement a 6.57 m^3^/s (150 Mgal/d) constant flow AWTF, in two phases. The AWTF will be located at the site of the Joint Water Pollution Control Plant (JWPCP) operated by the Sanitation Districts of Los Angeles County. Currently, the JWPCP has a capacity of 17.53 m^3^/s (400 Mgal/d) with an average daily flow of 11.39 m^3^/s (260 Mgal/d) (Chalmers et al., 2020). For the RRWR, a constant flow 1893 m^3^/d (0.5 Mgal/d) membrane bioreactor (MBR) is being piloted as the pretreatment step for the AWTF which will include reverse osmosis (RO) followed by ultraviolet light advanced oxidation (Chalmers et al., 2020). Whether the MBR is considered as part of the wastewater treatment process or part of the AWTF is open to debate. Regardless of how it is viewed, the performance of the MBR will be enhanced with constant flow and no return flows.

## Benefits of constant flow for wastewater treatment

The principal benefits of constant flow for wastewater treatment include (1) economic and operational considerations, (2) improved performance and increased process stability, (3) reduced energy usage, (4) ability to predict performance, (5) ability to implement new biological treatment processes, (6) ability to optimize performance of existing facilities for the removal of specific constituents, and (7) the ability to assign pathogen log removal credits (LRCs) for secondary WWTFs producing effluent for potable reuse. The corresponding benefits for AWTFs are considered in the following section.

### Economic and operational considerations

Cost and operational considerations are important in the implementation of constant flow WWTFs. In the design of conventional WWTFs, provision must be made for handling flowrate variations through the treatment process. Typical peaking factors (peak to average flow) used in design vary from 1.4 to 4, depending on the size of the WWTF. Where constant flow is used, the treatment process can be sized according to the requirements of the AWTF, resulting in significant cost savings over conventional design. Constant flow plants are also much easier to instrument, monitor, and operate.

### Improved process performance and stability

Depending on how constant flow is achieved, improved process performance is achieved as a result of the utilization of the average design hydraulic retention times, enhanced primary sedimentation, concentration, and load dampening; dilution of toxic constituents; reduction of shock loadings; enhanced secondary settling; and improved chemical feed application where employed. All of these measures are important in producing a high‐quality effluent for advanced water treatment (Tchobanoglous et al., 2014; WEF/ASCE, 2010). The benefits of constant versus variable flow treatment are also well documented in the literature (Greenwood et al., 2002; Gujer & Erni, 1978; LaGrega & Keenan, 1974; Niku et al., 1981; Plósz et al., 2009; US EPA, 1974; Vijayan et al., 2017).

### Reduced energy usage

Constant flow and reduced variability in the influent BOD concentrations and mass loadings, result in less average and peak energy usage for treatment as compared to treatment under variable flow and mass loadings conditions (Leu et al., 2009). If constant flow PF is employed to further reduce the total suspended solids (TSS) and BOD, energy savings of 15%–30% are achievable, depending on the operation of the biological treatment process (Caliskaner et al., 2020; Leu et al., 2012). The potential energy savings associated with constant flow or PF may be as important as the benefits for AWT.

### Predictable treatment performance

With constant flow and limited variability in the constituent concentrations to be treated, the performance of a biological wastewater treatment process can be predicted and modeled using steady‐state treatment kinetics. The ability to predict performance by WWTFs for the removal of specific constituents based on the average quantity and quality makes it possible to optimize the performance of AWTFs (Greenwood et al., 2002; Gujer & Erni, 1978).

### Implementation of new biological treatment processes

An exciting development in the field of environmental engineering is the number of new biological treatment processes that have been or are being developed (e.g., granular activated sludge, activate sludge with biocatalysts). Although each of the processes is different, they all share one common characteristic, a reduced volumetric requirement to achieve effective wastewater treatment (Farazaki & Gikas, 2019; de Kreuk & Loosdrecht, 2006). While the potential to reduce the tankage needed for treatment has space and cost implications, a significant problem exists with most new technologies in that constant flow operation plus or minus some small deviations is required. Thus, these new technologies, which will require some form of flow equalization, are ideally suited for use in divided treatment process (see Figure 4) and satellite treatment applications (see Figure 5). It has also been shown that membrane bioreactors, used for wastewater treatment, preform best when constant flow is maintained (Tchobanoglous et al., 2014).

### Removal of specific constituents

In addition to the removal of BOD and TSS, constant flow WWTFs can be combined with a number of sidestream bioaugmentation and treatment processes for the removal of specific constituents [e.g., nutrients, trace organics, and constituents of emerging concern (CECs)] (Babcock et al., 1992; Figdore et al., 2018; Leu et al., 2012; Nzila et al., 2016; Tchobanoglous et al., 2014). As new compounds and CECs are identified in the future, sidestream treatment and enhancement processes will become more important. The ability to operate at constant flowrate and with PF at essentially constant TSS loading opens up the possibility of implementing a number of new treatment processes that have been developed in the laboratory under steady‐state conditions.

### The ability to assign pathogen log removal credits (LRCs)

Potable water reuse projects must consider the removal of virus, *Giardia* cysts, and *Cryptosporidium* oocysts. In California, the LRCs required for the entire treatment process (i.e., WWTF plus AWTF) are: 12 log virus, 10 log *Giardia* cysts, and 10 log *Cryptosporidium oocysts*. To be able to assign LRCs for the removal of these microorganisms, the performance of the credited unit process must be stable and predictable. Where WWTFs are operated with variable flow, assignment of LRC for wastewater treatment can be penalized due to variation in treatment efficiency.

From a careful consideration of all of the possible removal mechanisms for *Giardia* cysts and *Cryptosporidium* oocysts, it is proposed that the principal mode of removal of these microorganisms in the activated sludge process is similar to their removal in water treatment facilities. In water treatment facilities, these organisms are removed through coagulation, flocculation, sedimentation, and filtration. The corresponding mechanisms in the activated sludge process are sorption on extracellular biological polymers during the conversion of organic matter to cell tissue, enmeshment in the biomass during aeration, sedimentation, and die‐off. Where primary sedimentation is employed there will also be some removal of particle‐associated cysts and oocysts during the sedimentation process. Two important observations from early studies on the removal *Giardia* cysts and *Cryptosporidium* oocysts in WWTFs were: (1) that the removal of the *Giardia* cysts was significantly greater than that for *Cryptosporidium* oocysts and (2) most of the cysts and oocysts were removed in secondary treatment process (Fu et al., 2010; Robertson et al., 2000).

**Figure 7:**
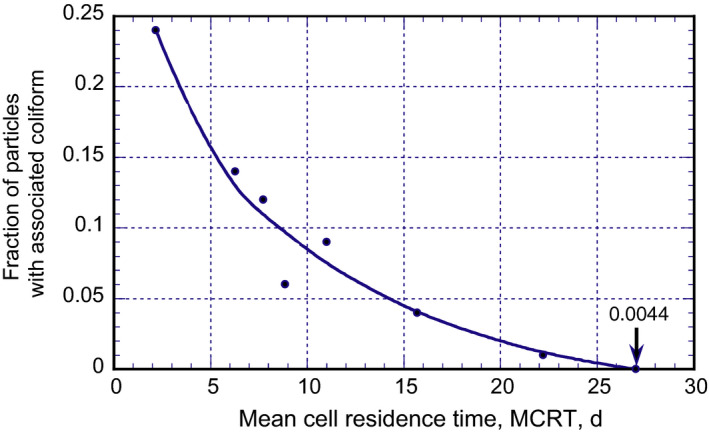
Plot of the fraction of particles with one or more associated coliform organisms as a function of the mean cell residence time (Adapted from Darby et al., 1999).

The enmeshment of microorganisms (coliform) associated with particles as a function of the mean cell residence time (MCRT) is shown in Figure 7. As shown in Figure 7, particles are less likely to contain coliform organisms with increasing MCRT. Die‐off occurs because some of the sorbed microorganisms are recycled in the return sludge until they become inactive. The actual removal achieved is based on the amount of settled activated sludge returned to the process and the amount of sludge wasted from the system. In turn, the amount of sludge wasted is a function of the process operating parameters, principally the MCRT. It should be noted that the same removal mechanisms are operative in the variable flow mode of operation, but the removal is variable and difficult to predict with any certainty. Thus, for a constant flow WWTF, the removal of these microorganisms is relatively constant and will depend on the mode of operation. Additionally, because of the consistency in sludge wasting at constant flow, the MCRT value achieved is also more consistent.

## Benefits of constant flow and optimized WWT for advanced water treatment

The benefits of an optimized treated effluent and constant flow on the operation and performance of AWTFs is considered in the following discussion. The benefits of maximizing the amount of flow that can be required are also considered.

### Benefits of effective constant flow wastewater treatment

The principal issue related to the use of existing WWTFs for the production of an effluent suitable for further processing in AWTFs is that they were designed to produce an effluent suitable for dispersal to the environment and not specifically for advanced treatment. With constant flow WWTF, an effluent can be produced that is optimized for AWT. For example, it has been demonstrated the changing the mode of operation of the biological process to produce a nitrified effluent will reduce the microfiltration membrane fouling significantly resulting in extended filter runs and reduced cleaning costs (Tchobanoglous et al., 2015; Voutchk, 2013). Many more opportunities are available for synergy between WWT and AWT. It is anticipated that integrated design between these treatment facilities will become common practice.

### Benefits of maximizing wastewater recovery for potable reuse

In water short areas, where the WWTFs are subject to large diurnal variations, the recovery of the maximum amount of water through flow equalization may be of greater importance than the treatment benefits. In parts of the country, the options for developing new water supplies are limited and include desalination of brackish groundwater, where available, or seawater, and imported water. Few, if any, sources of untapped water exist for importation. Water conservation is often incorrectly listed as a water source. The cost of advance treated water for potable reuse will, depending on the size of the installation, vary from about 0.65 to $1.30/m^3^ (800 to $1600/AF) without conveyance and RO brine management costs. By comparison, for large installations, the cost of desalination without conveyance costs will vary from 1.22 to $1.95/m^3^ (1500 to $2400/AF) (Raucher & Tchobanoglous, 2014). For small and medium sized installations, desalination costs can be up to three times greater. In the example shown in Figure 1a, if a flow of 0.066 m^3^/s (1.5 Mgal/d) could be saved though flow equalization and the market value of the water is $0.32/m^3^ ($400/AF) overproduction cost, the annual value of the recovered water is about $670,000. Depending on local construction costs and the type of flow equalization facilities employed, the payback period could be on the order of 3–4 years. As the value of water continues to increase, the economic benefits of flow equalization will become even more significant.

### Benefits of constant flow effluent pretreatment

Where constant flow is achieved through effluent diversion, but the treatment process has not been optimized to produce the highest quality effluent possible for AWT, additional pretreatment may be required to optimize the performance of the AWTF. A variety of different processes have been used including MBRs as a replacement for microfiltration; ozonation; and nitrifying biological aerated filters. In some locations where constant flow wastewater treatment has been implemented and optimized, it may still be desirable to provide additional pretreatment to enhance the overall performance of the AWTF. An additional pretreatment step that has been used is ozone coupled with biologically activated carbon. In all cases, all of these pretreatment processes benefit by being operated at a constant flowrate.

### Economic benefits of constant flow for capital and operation and maintenance costs

In general, AWTFs are sized and built in a modular size consistent with current and future potable reuse requirements. With constant flow and modular design, treatment costs for excess capacity are eliminated. As with WWTFs, constant flow AWTFs are also much easier to instrument, monitor, and operate. Constant flow coupled with effective pretreatment is also important in reducing wear and minimizing operational maintenance costs.

### Benefits of constant flow for process operation and maintenance

Constant flow is important for most treatment processes used for AWT. For example, reverse osmosis (RO) membranes have limited turndown and are typically operated within a narrow flow range. In an AWTF installation, there may be a number of RO membrane trains are employed. When there is insufficient flow to operate within specified flow ranges, an RO train may need to be taken out of service. As the flow increases, an out of service train would be brought back online. Constant flow minimizes the need to cycle RO banks, reduces wear, and simplifies operation. Similarly, UV based advanced oxidation facilities typically operate more effectively with limited flow variability. Recognizing the importance of constant flow for AWT and the need to recover treated wastewater which had to be discharged because of capacity limitation during the daytime hours, the Orange County Water District incorporated off‐line flow equalization facilities [see Figure 5c] as part of the Groundwater Replenishment System Final Expansion (Scott‐Roberts, 2016). One novel approach that has been implemented, for smaller flows, is to use an expandable membrane bladder. Bladders are available with capacities up to 950 m^3^ (250,000 gal).

### Benefit of pathogen LRCs from constant flow wastewater treatment

The value of receiving pathogen LRCs from wastewater treatment is in meeting the regulatory requirements or providing redundancy to meeting the requirements. Redundancy is important in meeting the LRV requirements for an AWTF in the situation where a process failure may occur. Perhaps, more importantly, redundancy is important in pubic outreach to assure the public that every possible precaution has been taken to protect public health and to maintain their confidence in potable reuse.

## Performance of two constant flow treatment facilities

To examine the proposition put forth in the previous section about the removal achieved in constant treatment facilities, the performance of two different constant flow treatment plants will be examined.

### Tillman WWTF, Los Angeles, CA

**Figure 8:**
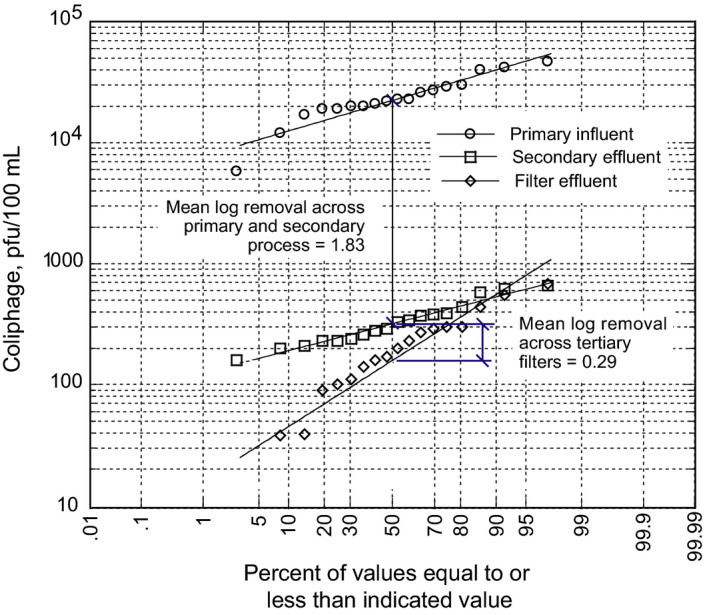
MS2‐Coliphage removal performance data from the early 2010s for the Tillman WWTF operated by the City of Los Angeles, CA. The Tillman WWTF, an extraction type satellite facility, is operated at a constant flowrate with no return flows. Comparing the influent and effluent coliphage plots, the removal through the process was essentially constant over a wide range of influent values, with some expected variation (Adapted from Tchobanoglous et al., 2014).

The Donald C. Tillman Water Reclamation Plant in the City of Los Angeles (see Figure 6), which operates as an extraction type satellite WWTF, was put into operation in 1985. Treated effluent from the plant is used to irrigate a world‐famous 2.6‐hectare Japanese garden, to fill the 1.11‐hectare lake located within the garden, and to maintain flow in the Los Angeles River. Studies are currently underway to co‐locate an AWTF to produce advanced treated effluent which will be used to augment the local groundwater through surface spreading at the Hansen spreading basins, located some 16 km (10 mi) away. Data from early 2010s for the removal of coliphage are illustrated on Figure 8. As shown, for the given operating conditions a mean log removal of 1.83 was achieved. Another way to think about the coliphage data is as follows. If a constant value of 1.83 log was subtracted from each of the influent coliphage values and the resulting values were plotted they would fall almost directly on the observed effluent distribution. As expected, some variability exists at the 5 and 95 percent probability values (1.77 log at 5 percent and 1.89 log at 95 percent). The corresponding minimum LRC value for this treatment process for the removal of coliphage would be 1.77 log. The observed variability can also be reflected in the confidence or prediction interval.

Also shown in Figure 8, is the coliphage removal achieved through the effluent filters. It is clear that the filter removal distribution does not follow the same distribution as the removal through the biological treatment process. In filtration, the pore size of the filtering medium changes from the start of a filter run to the end of the filter run, as material is removed within the pore space on the filter media. As a result, there are changes in the effectiveness of the operative removal mechanisms. Because there is variability throughout a filter run, the filter does not operate under steady‐state conditions. Thus, the removal achieved at the beginning and end of a filter run is not constant and will exhibit greater variability.

### North City Water Reclamation Plant, San Diego, CA

The North City Water Reclamation Plant (NCWRP) in San Diego, also an extraction type satellite plant which operates under constant primary effluent flow, was built initially to enhance local reuse of the treated effluent, primarily for irrigating golf courses. The NCWRP is currently the centerpiece of the Pure Water San Diego Program. Filter effluent from this facility will be treated further in the North City Pure Water Facility (NCPWF). Effluent from the NCPWF will be added to Miramar Reservoir to augment the local potable water supply. Water from the Miramar Reservoir will be blended with other water and treated in the Miramar Water Treatment Facility before being added to the water distribution network.

Currently, the NCWRP treatment process includes coarse screening, aerated grit removal, primary sedimentation, primary effluent flow equalization, activated sludge treatment, effluent filtration, and chlorine disinfection. The activated sludge process is operated as a modified Ludzack‐Ettinger process. There are no return flows to the treatment process. The average design flow was 1.31 m^3^/s (30 Mgal/d). The existing plant will be expanded to produce 1.84 m^3^/s (42 Mgal/d) for further treatment at the NCPWF. The activated sludge process is operated at a MCRT of 10 days. Representative performance data for the NCWRP for removal of microorganisms of concern are considered below.

**Figure 9:**
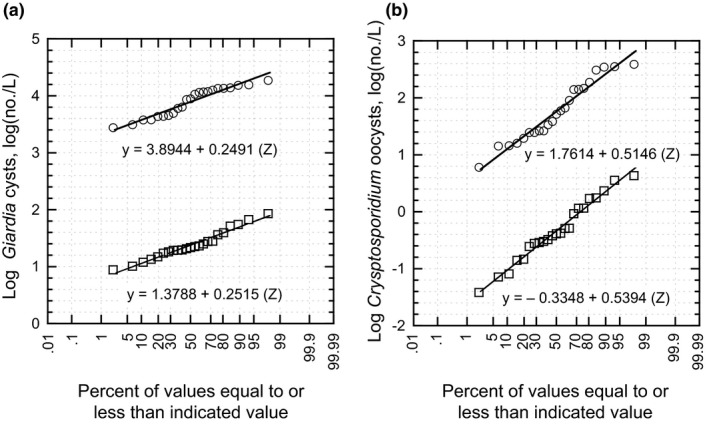
*Giardia* cysts and *Cryptosporidium* oocysts removal performance data for the North City Water Reclamation Plant (NCWRP) for the period from April 3, 2019 through March 3, 2020: (a) *Cryptosporidium* oocysts and (b) *Giardia* cysts. The NCWRP is operated at a constant flowrate with no return flows. The operating average mean cell residence time (MCRT) is 10 days. (Data courtesy City of San Diego, CA)

Performance data for the NCWRP between April 3, 2019, through March 9, 2020, for the removal of *Giardia* cysts and *Cryptosporidium* oocysts from the raw wastewater through the secondary effluent are shown in Figure 9a,b, respectively. As shown in Figure 9a,b, for the given operating conditions, the mean *Giardia* cyst and *Cryptosporidium* oocysts removal was 2.5156 log (3.8944–1.3788) and 2.0962 log [1.7614–(–0.3348)], respectively. The equation for the LRC probability distribution for cysts and oocysts is determined as the difference between two correlated random normal variables using the follow expression.

(1) $$I‐E∼N\left[\mu_{I}‐\mu_{E},\text{var}\left(I\right)+\text{var}\left(E\right)‐2\text{cov}\left(I,E\right)\right]$$

where *I*, *E* = influent and secondary effluent normal distributions, respectively. *µ_I_* = mean of influent distribution. *µ_E_* = mean of secondary effluent distribution. var(*I*) = variance of influent distribution. var(*E*) = variance of secondary effluent distribution. cov (*I*, *E*) = covariance in the *I* and *E* distributions.

Assuming rank pairing of the data presented on Figure 9, the covariance values for *Giardia* cysts and *Cryptosporidium* oocysts are 0.0598 and 0.2788, respectively. The resulting LRC probability distributions for *Giardia* cysts and *Cryptosporidium* oocysts are:

(3) $${\text{LRC}}_{\text{Co}}=2.0962+0.1085\left(Z\right)$$

where *Z* = value of standardized normal distribution for a given probability value.

Currently, the Division of Drinking Water (DDW) of the California State Water Resources Control Board only allows LRC that are achievable at 5 percent probability value. The removal of *Giardia* cysts at a probability value of 5 percent [where (*Z*) = −1.645] is equal to 2.34 log as given by the following expression.

$${\text{LRC}}_{0.05}=2.5156+0.1070\left(‐1.645\right)=2.340$$

The corresponding expression for Cryptosporidium oocysts is 1.919 log.

$${\text{LRC}}_{0.05}=2.0962+0.1085\left(‐1.645\right)=1.919$$

For both *Giardia* cysts and *Cryptosporidium* oocysts the removal through the treatment process is essentially constant, which is reflective of the stable operating conditions. If the MCRT is altered, changes in the log removal values would be anticipated. Based on the stable and consistent operation of the NCWRP treatment process, LRC values through secondary treatment of 2.34 and 1.92 log for *Giardia* cysts and *Cryptosporidium* oocysts*,* respectively, are consistent with the performance data. The ability to document the removal of *Giardia* cysts and *Cryptosporidium* oocysts is of significance not only with respect the overall LRC required by regulatory agencies, but it is also reassuring to the public that the most effective treatment possible is being provided.

## Discussion

Historically, WWTFs have been operated under conditions of variable flow with variable constituent loadings. However, with the implementation of satellite constant flow WWTFs in the City of Los Angeles, the Sanitation Districts of Los Angeles County, and the City of San Diego, the performance benefits of constant flow operation are well documented. It has been demonstrated that with constant flow operation, it is possible to assign LRCs with greater certainty of their reliability and reproducibility. The same benefits can be achieved with divided treatment. In effect, a divided constant flow treatment train can be considered the same as a satellite treatment facility. To achieve the highest quality effluent for advanced processing to produce potable water, constant flow WWTFs without any return flows offer significant performance benefits. Where constant flow is achieved by effluent diversion from conventional WWTFs additional pretreatment will be required.

Further improvements in the performance of constant flow WWTFs can be achieved with the application of new technologies such as PEF and/or PF. If new treatment processes are to be implemented, many of which will perform best under constant flow conditions, the conventional approach to the design of wastewater treatment facilities must be rethought, including the provision of flow equalization. In light of climate change, water supply stresses, and the need to reuse water an integrated water management strategy employing constant flow satellite WWTFs should be considered to produce the highest quality effluent for advanced water treatment.

Finally, it should be recognized that not all WWTFs which provide effluent for AWT for potable will be able or are willing to implement flow equalization. Reasons for not implementing flow equalization include the cost of equalization facilities, whether lined earthen basins or engineered tanks are used; space limitations for flow equalization facilities, either on‐site or off‐site; the cost‐effectiveness of flow equalization for achieving stable performance and additional pathogen LRCs versus obtaining additional LRCs through the AWTF; and the assumption that the design of the AWTF should be able to deal with less than optimum variable secondary effluent quality.

In some locations, the AWTF may only treat a portion of the total flow, in which case additional flow equalization facilities may not be needed, but additional pretreatment may be required. In other locations, it may be more cost‐effective to implement flow equalization when a new AWTF is being built. Where wastewater flow rates are decreasing, due to water conservation, and no other nearby sources are available it may be necessary to implement flow equalization to both recover any excess water that may have to be wasted during the day and to maintain constant flow through the AWTF.

## Summary

The advantages of constant flow wastewater treatment with no return flows for the production of treated effluent of the highest quality for further processing in an advanced water treatment facility have been presented and discussed. Constant flow can be achieved by flow equalization, the use of divided treatment trains, and satellite treatment. With existing WWTFs flow equalization may be difficult to implement due to space limitations and cost considerations. In some locations, the decrease in the available amount of wastewater due to water conservation many necessitate the use of flow equalization facilities. In the future, to more effectively utilize the available wastewater, satellite wastewater treatment will become more common. Predictable LRCs can be allocated for *Giardia* cysts and *Cryptosporidium* oocysts in constant flow WWTFs. As new compounds are identified, integrated design between WWTFs and AWTFs will be needed to achieve optimal treatment that is protective of public health.

## Author contributions

**George Tchobanoglous**: Conceptualization (equal); Writing‐original draft (lead); Writing‐review & editing (equal). **John Kenny**: Data curation (equal); Writing‐review & editing (equal). **Harold Leverenz**: Conceptualization (equal); Visualization (equal); Writing‐review & editing (equal).
